# Supplementary material for: Interferon Beta Activity Is Modulated via Binding of Specific S100 Proteins
Source: Int J Mol Sci. 2020 Dec 13;21(24):9473. doi: 10.3390/ijms21249473 (PMC7764042; doi:10.3390/ijms21249473)
Supplement: Supplementary file 1 [file ijms-21-09473-s001.pdf]

Supplementary Materials

Interferon beta activity is modulated via binding of specific S100 proteins

Alexey S. Kazakov [1], Alexander D. Sofin [1], Nadezhda V. Avkhacheva [1], Alexander I. Denesyuk [1,2], Evgenia I. Deryusheva [1], Victoria A. Rastrygina [1], Andrey S. Sokolov [1], Maria E. Permyakova [1], Ekaterina A Litus [1], Vladimir N. Uversky [1,3,*], Eugene A. Permyakov [1], and Sergei E. Permyakov [1,*]

[1] Institute for Biological Instrumentation, Pushchino Scientific Center for Biological Research of the Russian Academy of Sciences, Pushchino, Moscow region, 142290 Russian Federation; fenixfly@yandex.ru (ASK), AlSofin@mail.ru (ADS), avkhacheva@gmail.com (NVA), adenesyu@abo.fi (AID), janed1986@ya.ru (EID), certusfides@gmail.com (VAR), 212sok@gmail.com (ASS), mperm1977@gmail.com (MEP), ealitus@gmail.com (EAL), epermyak@yandex.ru (EAP), permyakov.s@gmail.com (SEP)

[2] Structural Bioinformatics Laboratory, Biochemistry, Faculty of Science and Engineering, Åbo Akademi University, Turku, 20520 Finland; adenesyu@abo.fi

[3] Department of Molecular Medicine and USF Health Byrd Alzheimer's Research Institute, Morsani College of Medicine, University of South Florida, Tampa, FL, USA; vuversky@usf.edu

* Correspondence: Dr. Sergei E. Permyakov, Institute for Biological Instrumentation, Pushchino Scientific Center for Biological Research of the Russian Academy of Sciences, Institutskaya str., 7, Pushchino, Moscow region, 142290 Russia; Email: permyakov.s@gmail.com; Tel: +7 (495) 143-7741; Fax: +7(4967) 33 05 22; Prof. Vladimir N. Uversky, Department of Molecular Medicine and USF Health Byrd Alzheimer's Research Institute, Morsani College of Medicine, University of South Florida, Tampa, FL 33612, USA; Email:vuversky@usf.edu

Figure S1. Multiple sequence alignment of human S100 proteins analyzed in this study using the Clustal Omega algorithm (**A**), Percent Identity Matrix (**B**), and corresponding evolutionary tree (**C**).

A

```
CLUSTAL O(1.2.4) multiple sequence alignment


sp|P25815|S100P_HUMAN      --------------MTELETAMGMIIDVFSRYSGSEGSTQTLTKGELKVLMEKELPGFLQ        46
sp|P23297|S10A1_HUMAN      -------------MGSELETAMETLINVFHAHSGKEGDKYKLSKKELKELLQTELSGFLD        47
sp|P26447|S10A4_HUMAN      -------------MACPLEKALDVMVSTFHKYSGKEGDKFKLNKSELKELLTRELPSFLG        47
sp|P06703|S10A6_HUMAN      -------------MACPLDQAIGLLVAIFHKYSGREGDKHTLSKKELKELIQKELT--IG        45
sp|P04271|S100B_HUMAN      --------------MSELEKAMVALIDVFHQYSGREGDKHKLKKSELKELINNELSHFLE        46
sp|P31151|S10A7_HUMAN      ------------MSNTQAERSIIGMIDMFHKYTRRDD---KIEKPSLLTMMKENFPNFLS        45
sp|P05109|S10A8_HUMAN      -------------MLTELEKALNSIIDVYHKYSLIKGNFHAVYRDDLKKLLETECPQYIR        47
sp|P06702|S10A9_HUMAN      ----------MTCKMSQLERNIETIINTFHQYSVKLGHPDTLNQGEFKELVRKDLQNFLK        50
sp|P60903|S10AA_HUMAN      -------------MPSQMEHAMETMMFTFHKFAGDKG---YLTKEDLRVLMEKEFPGFLE        44
sp|P31949|S10AB_HUMAN      --------MAKISSPTETERCIESLIAVFQKYAGKDGYNYTLSKTEFLSFMNTELAAFTK        52
sp|P80511|S10AC_HUMAN      --------------MTKLEEHLEGIVNIFHQYSVRKGHFDTLSKGELKQLLTKELANTIK        46
sp|Q99584|S10AD_HUMAN      ---------MAAEPLTELEESIETVVTTFFTFARQEGRKDSLSVNEFKELVTQQLPHLLK        51
sp|Q9HCY8|S10AE_HUMAN      MGQCRSANAEDAQEFSDVERAIETLIKNFHQYS-VEGGKETLTPSELRDLVTQQLPHLMP        59
sp|Q86SG5|S1A7A_HUMAN      ------------MSNTQAERSIIGMIDMFHKYTGRDG---KIEKPSLLTMMKENFPNFLS        45
                                             :  :  ::  :  .:   .    :   .:  ::  :

sp|P25815|S100P_HUMAN      SG--KDKDAVDKLLKDLDANGDAQVDFSEFIVFVAAITSACHKYFEKAGLK---------        95
sp|P23297|S10A1_HUMAN      AQ--KDVDAVDKVMKELDENGDGEVDFQEYVVLVAALTVACNNFFWENS-----------        94
sp|P26447|S10A4_HUMAN      KR--TDEAAFQKLMSNLDSNRDNEVDFQEYCVFLSCIAMMCNEFFEGFPDKQPRKK----        101
sp|P06703|S10A6_HUMAN      SK--LQDAEIARLMEDLDRNKDQEVNFQEYVTFLGALALIYNEALKG-------------        90
sp|P04271|S100B_HUMAN      EI--KEQEVVDKVMETLDNDGDGECDFQEFMAFVAMVTTACHEFFEHE------------        92
sp|P31151|S10A7_HUMAN      ACDKKGTNYLADVFEKKDKNEDKKIDFSEFLSLLGDIATDYHKQSHGAAPCSGGSQ----        101
sp|P05109|S10A8_HUMAN      KK------GADVWFKELDINTDGAVNFQEFLILVIKMGVAAHKKSHEESHKE--------        93
sp|P06702|S10A9_HUMAN      KEN-KNEKVIEHIMEDLDTNADKQLSFEEFIMLMARLTWASHEKMHEGDEGPGHHHKPGL        109
sp|P60903|S10AA_HUMAN      NQ--KDPLAVDKIMKDLDQCRDGKVGFQSFFSLIAGLTIACNDYFVVHMKQKGKK-----        97
sp|P31949|S10AB_HUMAN      NQ--KDPGVLDRMMKKLDTNSDGQLDFSEFLNLIGGLAMACHDSFLKAVPSQKRT-----        105
sp|P80511|S10AC_HUMAN      NI--KDKAVIDEIFQGLDANQDEQVDFQEFISLVAIALKAAHYHTHKE------------        92
sp|Q99584|S10AD_HUMAN      DV-----GSLDEKMKSLDVNQDSELKFNEYWRLIGELAKEIRKKK----DLKIRKK----        98
sp|Q9HCY8|S10AE_HUMAN      SN-----CGLEEKIANLGSCNDSKLEFRSFWELIGEAAKSVKLER----PVRGH------        104
sp|Q86SG5|S1A7A_HUMAN      ACDKKGIHYLATVFEKKDKNEDKKIDFSEFLSLLGDIAADYHKQSHGAAPCSGGSQ----        101
                                        :   .   *    * .:  ::       .

sp|P25815|S100P_HUMAN      -----   95
sp|P23297|S10A1_HUMAN      -----   94
sp|P26447|S10A4_HUMAN      -----   101
sp|P06703|S10A6_HUMAN      -----   90
sp|P04271|S100B_HUMAN      -----   92
sp|P31151|S10A7_HUMAN      -----   101
sp|P05109|S10A8_HUMAN      -----   93
sp|P06702|S10A9_HUMAN      GEGTP   114
sp|P60903|S10AA_HUMAN      -----   97
sp|P31949|S10AB_HUMAN      -----   105
sp|P80511|S10AC_HUMAN      -----   92
sp|Q99584|S10AD_HUMAN      -----   98
sp|Q9HCY8|S10AE_HUMAN      -----   104
sp|Q86SG5|S1A7A_HUMAN      -----   101
```

B

```
#
#
#  Percent Identity  Matrix - created by Clustal2.1
#
#
```

sp|P25815|S100P_HUMAN	100.00	52.69	41.05	34.83	50.00	27.17	36.26	35.79	43.48	38.95	44.57	26.14	24.14	29.35
sp|P23297|S10A1_HUMAN	52.69	100.00	48.94	43.33	57.61	20.88	37.78	36.17	47.25	40.43	36.96	34.48	25.58	23.08
sp|P26447|S10A4_HUMAN	41.05	48.94	100.00	51.11	45.65	22.45	29.03	27.72	34.02	28.00	32.61	32.98	25.27	24.49
sp|P06703|S10A6_HUMAN	34.83	43.33	51.11	100.00	39.33	22.99	27.91	30.00	26.44	28.89	34.83	30.59	26.19	25.29
sp|P04271|S100B_HUMAN	50.00	57.61	45.65	39.33	100.00	25.84	34.09	36.96	37.08	34.78	38.04	26.74	28.24	26.97
sp|P31151|S10A7_HUMAN	27.17	20.88	22.45	22.99	25.84	100.00	24.44	24.00	22.68	32.65	25.84	19.57	21.35	94.06
sp|P05109|S10A8_HUMAN	36.26	37.78	29.03	27.91	34.09	24.44	100.00	25.81	27.78	26.88	39.77	22.47	18.18	25.56
sp|P06702|S10A9_HUMAN	35.79	36.17	27.72	30.00	36.96	24.00	25.81	100.00	29.90	32.04	46.74	30.93	29.79	25.00
sp|P60903|S10AA_HUMAN	43.48	47.25	34.02	26.44	37.08	22.68	27.78	29.90	100.00	36.08	31.46	26.67	27.27	24.74
sp|P31949|S10AB_HUMAN	38.95	40.43	28.00	28.89	34.78	32.65	26.88	32.04	36.08	100.00	35.87	32.99	25.00	34.69
sp|P80511|S10AC_HUMAN	44.57	36.96	32.61	34.83	38.04	25.84	39.77	46.74	31.46	35.87	100.00	32.56	27.06	26.97
sp|Q99584|S10AD_HUMAN	26.14	34.48	32.98	30.59	26.74	19.57	22.47	30.93	26.67	32.99	32.56	100.00	36.84	19.57
sp|Q9HCY8|S10AE_HUMAN	24.14	25.58	25.27	26.19	28.24	21.35	18.18	29.79	27.27	25.00	27.06	36.84	100.00	22.47
sp|Q86SG5|S1A7A_HUMAN	29.35	23.08	24.49	25.29	26.97	94.06	25.56	25.00	24.74	34.69	26.97	19.57	22.47	100.00

C

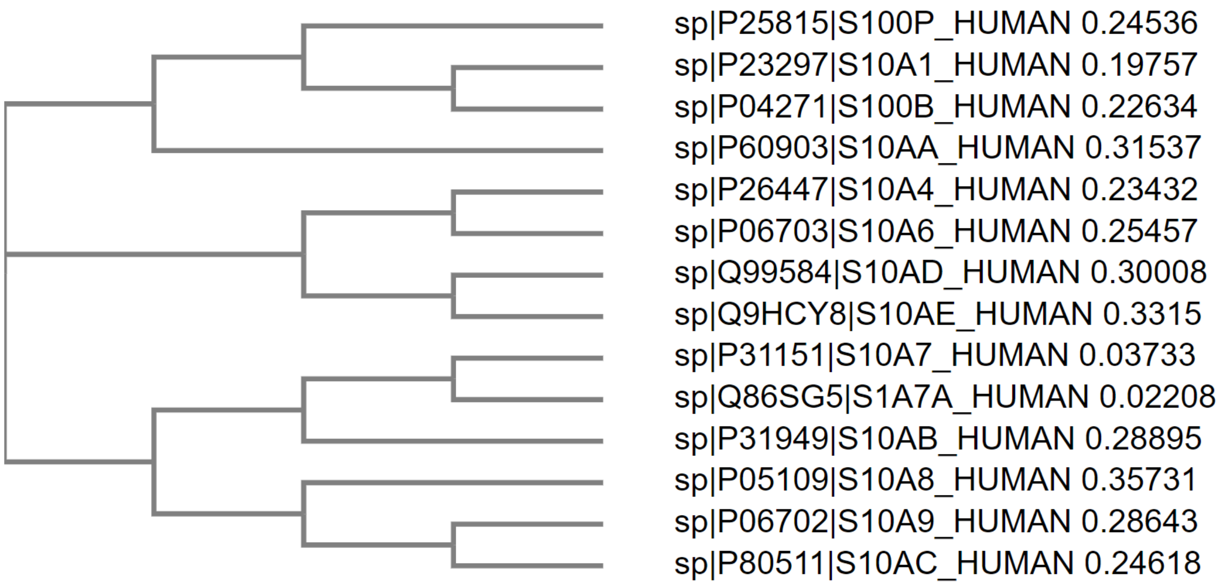

Supplementary files

Protein Data Bank files of the models of tertiary structures of INF-β complexes with S100A1/A4/A6/P proteins (**Figure 9**), constructed as described in *Materials and Methods* section.

Model of S100A1-IFNb complex.pdb

Model of S100A4-IFNb complex.pdb

Model of S100A6-IFNb complex.pdb

Model of S100P-IFNb complex.pdb

Table S1. Molar extinction coefficients at 280 nm for the proteins used in the present study, calculated according to ref. (Pace, Vajdos et al. 1995).

Protein	ε_{280nm}, $M^{-1}cm^{-1}$
IFN-β	31,525
IFN-α	18,700
S100A1	8,480
S100A4<br>S100A10<br>S100A12	2,980
S100A6<br>S100A7<br>S100A11<br>S100A15	4,470
S100A8	11,460
S100A9<br>S100A13<br>S100A14	6,990
S100B	1,490

Table S2. List of the human diseases associated with IFN-β and S100A1 protein, according to DisGeNET database (http://www.disgenet.org). PubMed identifiers of the references confirming the protein-disease associations are indicated.

№	*Disease*	*IFN-β*	*S100A1*
1	Cardiomyopathy, Dilated	20350703	22336719
2	Encephalopathies	8615598	28072884
3	Vitiligo	28752785	15095274
4	Tumor Cell Invasion	29618645; 28317173; 25486572	30216200; 22834835; 20819668
5	Malignant neoplasm of ovary	11896621	31033462; 30558666; 15277215
6	Non-Small Cell Lung Carcinoma	30622322; 26431376; 23658645	29607329; 17689067
7	Coronary Artery Disease	25882064	31523180; 26515415
8	Brain Diseases	12618863; 8615598	30392897
9	Neoplasms	31512776; 31776268; 30683475; 31070806; 31627888; 30848981; 31036052; 30393160; 31358054; 31431457; 30675668; 30679806; 30622322; 29750542; 30012853; 29849115; 28912136; 28337378; 28514874; 27188205; 28578991; 28098415; 28483787; 27399807; 26880763	30710303
10	Primary malignant neoplasm	30864696; 30613977; 31167402; 31348591; 30679806; 31036052; 30446226; 29750542; 29158470; 27637889; 29229854; 28214639; 28912136; 28129467; 26597133; 27077807; 26880763; 27496321; 27532609; 24141111; 23403306; 22522623; 22842821; 21226949; 22106296	30710273; 28595036; 25614008; 19926575; 17523080; 15336958; 12445462
11	Malignant Neoplasms	30864696; 30679806; 31348591; 31036052; 31167402; 30613977; 30446226; 29158470; 29750542; 28214639; 26597133; 29229854; 28912136; 28129467; 27637889; 27532609; 26880763; 27496321; 27077807; 24141111; 23403306; 22522623; 22842821; 22106296; 21226949	30710273; 28051137; 28595036; 25614008; 19926575; 17523080; 15336958
12	Renal Cell Carcinoma	22344395; 18695887; 15546502; 12631618	25597876; 19680475; 17394501; 17483815; 17483815; 11734338
13	Malignant neoplasm of urinary bladder	11948141; 2297754	17970044

14	Malignant neoplasm of stomach	22159640	30414696; 25266115; 22295074; 18793447; 17400182
15	Amyloidosis	30222725	24931125
16	Malignant neoplasm of lung	30683475; 30622322; 30446226; 26351076; 25486572; 19893592; 19564073; 2560625	29146477
17	Tumor Progression	31821828; 28849002	21153724
18	Malignant neoplasm of pancreas	23807450	12950018
19	Conventional (Clear Cell) Renal Cell Carcinoma	22344395; 18695887; 15546502; 12631618	11734338
20	Secondary malignant neoplasm of lymph node	30672717; 23358428	28595036
21	Alzheimer's Disease	30610591; 31152860; 31233762; 30076830; 30222725; 24262201	31156365; 31079281; 24931125
22	Malignant neoplasm of breast	31167402; 26716512; 27077807; 24141111; 18608205; 18608205	30018736; 28051137; 21153724
23	Coronary heart disease	25882064	31523180
24	Liver carcinoma	28456632; 23887606; 22790964; 19911974; 18618506; 18083266; 12395321	29901195
25	Squamous cell carcinoma of esophagus	23639140; 21452064	15185146
26	Bladder Neoplasm	11948141; 2297754	17523080; 17970044
27	Breast Carcinoma	31167402; 27207649; 27077807; 26716512; 25884794; 24141111; 18608205; 18608205; 9059994	30018736; 28051137; 21153724; 16273201
28	Neoplasm Metastasis	31070806; 30213181; 29202279; 28129467; 26716512; 26102027; 20197756; 12405292; 11948141; 11498771; 10029078; 9815626	15608682

Table S3. List of the human diseases associated with IFN-β and S100A1 protein, according to Open Targets Platform database (https://www.opentargets.org), and corresponding association scores (https://docs.targetvalidation.org/getting-started/scoring). Diseases with association scores for the both proteins exceeding 0.1 are highlighted in yellow.

№	*Disease*	*IFN-β*	*S100A1*
1	amyloidosis	0.013	0.024
2	weight loss	0.022	0.025
3	vasculitis	0.043	0.024
4	chronic obstructive pulmonary disease	0.087	0.012
5	psoriasis	0.056	0.02
6	hepatocellular carcinoma	0.112	0.073
7	gastric adenocarcinoma	0.026	0.363
8	diarrheal disease	0.035	0.024
9	inflammation	0.102	0.061
10	urethral intrinsic sphincter deficiency	0.043	0.018
11	arthritis	0.13	0.028
12	idiopathic pulmonary fibrosis	0.031	0.193
13	atherosclerosis	0.052	0.015
14	glioblastoma multiforme	0.676	0.031
15	neuroblastoma	0.082	0.01
16	cancer	0.839	0.538
17	ovarian carcinoma	0.697	0.082
18	hyperplasia	0.051	0.018
19	urinary bladder cancer	0.06	0.049
20	breast cancer	0.716	0.046
21	medulloblastoma	0.042	0.062
22	shock	0.044	0.016
23	cervical cancer	0.047	0.036
24	hypoxia	0.046	0.064
25	infectious disease	0.295	0.198
26	nervousness	0.066	0.018
27	cardiomyopathy	0.283	0.192
28	renal cell adenocarcinoma	0.078	0.061
29	vascular disease	0.228	0.322
30	heart disease	0.283	0.312
31	cerebral infarction	0.045	0.011
32	myocardial infarction	0.024	0.07
33	endometrial carcinoma	0.061	0.031
34	colorectal carcinoma	0.063	0.011

35	hyperglycemia	0.012	0.016
36	atopic eczema	0.013	0.011
37	obesity	0.198	0.02
38	gastric carcinoma	0.042	0.367
39	ischemia reperfusion injury	0.023	0.026
40	clear cell renal carcinoma	0.024	0.013
41	neoplasm	0.85	0.586
42	acromegaly	0.038	0.01
43	malignant pancreatic neoplasm	0.058	0.011
44	breast adenocarcinoma	0.029	0.011
45	hypersensitivity reaction disease	0.261	0.044
46	substance dependence	0.062	0.027
47	metastatic melanoma	0.05	0.009
48	glioma	0.788	0.021
49	potassium-aggravated myotonia	0.044	0.018
50	anaplastic astrocytoma	0.045	0.011
51	juvenile idiopathic arthritis	0.011	0.013
52	cardiotoxicity	0.013	0.05
53	type I diabetes mellitus	0.048	0.039
54	primitive neuroectodermal tumor	0.083	0.021
55	Fanconi anemia complementation group E	0.014	0.015
56	complication	0.078	0.011
57	cardiac hypertrophy	0.011	0.041
58	Alzheimer's disease	0.044	0.04
59	dermatomyositis	0.071	0.02
60	hypertension	0.102	0.314
61	kidney neoplasm	0.199	0.066
62	nervous system disease	0.85	0.322
63	heart failure	0.015	0.303
64	dilated cardiomyopathy	0.257	0.02
65	necrosis	0.064	0.012
66	infertility	0.203	0.021
67	tongue neoplasm	0.043	0.01
68	pulmonary arterial hypertension	0.102	0.309
69	refractory drug response	0.025	0.012
70	non-small cell lung carcinoma	0.646	0.004
71	cardiac arrhythmia	0.031	0.026
72	stomach neoplasm	0.064	0.368
73	autoimmune retinopathy	0.01	0.01

74	cutaneous melanoma	0.038	0.02
75	cirrhosis of liver	0.061	0.2
76	nicotine dependence	0.06	0.013
77	dysplasia	0.029	0.017
78	squamous cell carcinoma	0.055	0.43
79	carcinoma	0.839	0.537
80	tetanus	0.033	0.011
81	diabetes mellitus	0.05	0.196
82	injury	0.075	0.036
83	ischemia	0.053	0.035
84	esophageal squamous cell carcinoma	0.04	0.01
85	glomerulonephritis (disease)	0.048	0.01
86	adenocarcinoma	0.783	0.383
87	lung carcinoma	0.646	0.424
88	myocarditis	0.049	0.012
89	ulcerative colitis	0.05	0.024
90	astrocytoma	0.676	0.015
91	uveal melanoma	0.037	0.024
92	sepsis	0.155	0.007
93	melanoma	0.141	0.047
94	lymph node metastatic carcinoma	0.029	0.037

Table S4. List of the human diseases associated with IFN-β and S100A4 protein, according to DisGeNET database (http://www.disgenet.org). PubMed identifiers of the references confirming the protein-disease associations are indicated.

№	*Disease*	*IFN-β*	*S100A4*
1	Cervix carcinoma	10521926	28921916
2	Cervical cancer	10521926	28921916
3	Malignant neoplasm of urinary bladder	11948141; 2297754	29218245; 26508026; 11839590
4	Pancreatic carcinoma	23807450	31526392; 29578167; 25677816; 24732359; 25072505; 23026527; 23085231; 19653048; 19799859; 18350635; 17786304; 11786397
5	Glioblastoma Multiforme	29557060; 29159280; 28912136; 28098415; 27637889; 27194146; 25920530; 26441059; 24526161; 23384727; 20606645; 19197327; 18714312; 14965379; 1653364; 1998958	28807938; 19074870
6	Respiratory Distress Syndrome, Adult	29365277	25070658
7	Renal Cell Carcinoma	22344395; 18695887; 15546502; 12631618	19365673
8	Crohn Disease	29358708	20489045
9	Prostate carcinoma	29435019; 26339361; 25075566; 23790156; 22429371; 16989575; 17088978; 12231540; 11498771	30041429; 27566074; 28341124; 17219414; 16990429
10	Breast Carcinoma	31167402; 27207649; 27077807; 26716512; 25884794; 24141111; 18608205; 18608205; 9059994	30450809; 31709177; 31667000; 29733962; 28058579; 27927689; 27098123; 26285644; 25205038; 25311085; 23543443; 23162804; 22740693; 21195708; 19055112; 15608682; 15956747; 12756252; 14632631; 12517790; 11875708; 11839590; 9751936; 9570150
11	Endometrial Carcinoma	27532609; 21226949; 21868529	29901152; 24756855; 19506550; 17673926
12	Inflammatory Bowel Diseases	27220814	30221056; 28935867
13	Chronic Obstructive Airway Disease	31170225; 31260505; 28379462; 22881993; 22543056	26483185
14	Idiopathic pulmonary arterial hypertension	31798832; 30639829	30031694; 23525442
15	Neoplasm Metastasis	31070806; 30213181; 29202279; 28129467; 26716512; 26102027; 20197756; 12405292; 11948141; 11498771; 10029078; 9815626	3146723;1 30885944; 31709177; 30927479; 31474194; 30745574; 30710284; 31775027; 30893511; 30410586; 29741811; 30221056; 29901152; 29251175; 29449540;

			29544454; 29789685; 29932233; 29733962; 29303514 ;30111999; 29800090; 28244731; 28058579; 27927689
16	Rheumatoid Arthritis	30291236; 26107769; 23369825; 22614743	31037071; 30710284; 19828600; 17328050; 17328050; 17105852
17	Triple-Negative Breast Carcinoma	31036052; 29750542; 29229854	28247948; 25667103
18	Melanoma	31356866; 30864696; 30213181; 31070806; 28649738; 27677689; 28624449; 28877249; 29136453; 27399807; 26102027; 26054674; 25728676; 23370279; 23358428; 23018621; 22555508; 21723255; 21846298; 21493591; 18945721; 16505900; 16928243; 16343929; 12096926	29679610; 26928771; 24613382; 9703888; 9291441
19	Adult Medulloblastoma	28559152; 24584142	17579622; 12517790
20	Psoriasis	27438769	30710284; 21712367
21	Hepatitis A	30594597; 22106296; 16937453; 16103148; 12414934	31321478; 28688902
22	Malignant neoplasm of esophagus	12168834	22912541; 22408350; 21889495
23	Malignant neoplasm of ovary	11896621	19194111
24	Childhood Medulloblastoma	28559152; 24584142	17579622; 12517790
25	Liver carcinoma	28456632; 23887606; 22790964; 19911974; 18618506; 18083266; 12395321	30897344; 27544906; 24065232; 23483190; 17051636
26	Ductal Breast Carcinoma	24141111	28223108
27	Carcinoma of bladder	11948141	29218245; 26508026; 11839590
28	Brain Neoplasms	23384727; 21805051; 19197327; 1501894	17223348
29	Malignant tumor of colon	31348591; 29496994; 18035482	30111999; 29932233; 21403839; 21685359; 21795396; 21685359; 21289293; 20515499; 17101323
30	Glioblastoma	29557060; 27637889; 28098415; 29159280; 28912136;27194146; 25920530; 26441059; 26397698; 24526161; 23384727; 21805051; 20606645; 19197327; 15455376; 14965379; 1501894; 1998958; 1653364	28807938; 25854377; 19074870; 17223348
31	Fatty Liver	29855540	31321478
32	Malignant neoplasm of pancreas	23807450	29578167; 25677816; 25072505; 24732359; 23026527; 23085231;

			19799859; 19653048; 18350635; 17786304; 12874021
33	Esophageal Neoplasms	12168834	22408350; 22912541; 21889495
34	Childhood Glioblastoma	29557060; 28098415; 29159280; 27637889; 27194146; 26441059; 25920530; 24526161; 19197327; 1998958	28807938
35	Nephroblastoma	25312909	24706305
36	Colon Carcinoma	31348591; 29496994; 18035482; 1586975	30111999; 29932233; 21795396; 21685359; 21289293; 21403839; 20515499; 17101323; 11875708
37	Mesothelioma	20379224; 20068553; 19773437	21983934
38	Pulmonary Fibrosis	28084646	29940125; 29910813; 30127784; 15618458
39	Astrocytoma	19267105; 15455376; 8174086; 8103050; 1906070	25854377
40	Lymphoma	22698399	7536040
41	Stomach Carcinoma	22159640	29342841; 30410586; 26497012; 25310523; 25511023; 19820999; 19945782; 12887505; 11106237
42	Adrenoleukodystrophy	31662417	31321478
43	Fibrosis, Liver	25715168	31321478; 28688902 30328321; 25111176 19506550
44	Bladder Neoplasm	11948141; 2297754	29218245; 26508026; 12532418; 11839590
45	Mammary Neoplasms	18608205	30450809; 17550972; 17550972; 16489073; 15856021; 14632631; 12439718; 10738249
46	Osteoporosis	25563300; 12944401	31814858
47	Malignant neoplasm of prostate	29435019; 26339361; 25075566; 23790156; 22429371; 17088978; 16989575; 12231540; 11498771	30041429; 28341124; 27566074; 17219414; 16990429
48	Leukemia	2295067	18452107
49	Obesity	29914624; 22951153	29789685
50	Medulloblastoma	28559152; 24584142	17579622; 17579622; 12517790; 12517790
51	Malignant Neoplasms	30864696; 30679806; 31348591; 31036052; 31167402; 30613977; 30446226; 29750542; 28214639; 26597133; 29229854; 28912136; 28129467; 27637889; 27532609; 26880763; 27496321; 27077807; 24141111; 23403306; 22522623; 22842821; 22106296; 21226949	30868407; 31474194; 30885944; 31445888; 30710284; 31623154; 30045697; 29556233; 29901152; 29069865; 28807938; 28530639; 28921916; 28935867; 28247948; 27098123; 27793047; 27127879; 27278086; 26497012; 24798676; 25171474; 24174222; 24039846
52	Glioma	31638255; 31100209; 26252165; 25175315; 24671169; 24526161; 22938469; 21805051; 19893595; 18220319; 16343929; 14965379;	28807938; 25854377; 7898625

		15546502; 11856487; 10982152; 10395172; 9875676; 1998958	
53	Carcinoma of lung	30622322; 31428903; 30683475; 30446226; 26351076; 25486572; 19893592; 19564073; 2560625	30885944; 27127879; 24732359; 9066685
54	Fever	20797409; 10395172	17497677; 9468217
55	Myositis	24757153; 21177291	21712367
56	Colorectal Neoplasms	12405292	16097057
57	Secondary Neoplasm	20197756	29800090; 29449540; 29901152; 27750112; 25205038; 25311085; 23483190; 22381352; 22878175; 23162804; 22292654; 21352421; 19820999; 19365673; 19194111; 19055112; 17101323; 16265347; 11839590; 11875708; 9696040; 8824556; 8968106; 7536040
58	Secondary malignant neoplasm of bone	29435019	31667000; 28615627
59	Malignant neoplasm of lung	30683475; 30622322; 30446226; 26351076; 25486572; 19893592; 19564073; 2560625	30885944; 27127879; 24732359
60	Childhood Astrocytoma	19267105; 8103050; 1906070	25854377
61	Asthma	31461630; 30950247; 31849956; 29611236; 28192616; 28415826; 27622317; 27238549; 27695127; 20627922	30569582
62	Secondary malignant neoplasm of lymph node	30672717; 23358428	31467231; 30868407; 29383839; 30111999; 28457731; 24756855; 25217321; 25171474; 23580256; 21603862; 23162804; 21837681; 22200787; 21218086; 19820999; 18360353; 17032501; 16265347; 16097057; 15579771; 12887505
63	Non-Small Cell Lung Carcinoma	26431376; 23658645	28197929; 27127879; 24215488; 22761930; 18620780; 8060323
64	Complete atrioventricular block	22992810	28688902
65	Malignant neoplasm of stomach	22159640	30410586; 29342841; 26497012; 25511023; 25310523; 21618249; 19945782; 19820999; 12887505; 11106237
66	Esophageal carcinoma	12168834	22408350; 22912541; 21889495
67	Triple Negative Breast Neoplasms	31036052; 29750542; 29229854	28247948;25667103
68	Leukemia, Myelocytic, Acute	17047156	31611628; 30466095
69	Adult Glioblastoma	29557060; 28098415; 29159280; 27637889; 27194146; 26441059;	28807938

		25920530; 24526161; 19197327; 1998958	
70	Neuroblastoma	27637889; 26887385; 28882441; 26943965; 25920530; 25086905; 18726131; 16115947	28687621; 15852272; 11431772
71	Childhood Neuroblastoma	26887385; 28882441; 27637889; 26943965; 25920530; 25086905; 18726131	28687621; 15852272; 11431772
72	Squamous cell carcinoma of the head and neck	25995245	31159419; 27793047; 24798676; 24037664
73	Malignant neoplasm of breast	31167402; 26716512; 27077807; 25884794; 24141111; 18608205; 18608205	30450809; 31667000; 31709177; 29733962; 27927689; 28058579; 27098123; 26285644; 25311085; 23543443; 22740693; 23162804; 21195708; 19055112; 15956747; 15608682; 14632631; 12517790; 12756252; 11839590; 11875708; 10738249; 9570150
74	Hypertensive disease	31798832	30031694; 16002749
75	Primary malignant neoplasm	30864696; 30613977; 31167402; 31348591; 30679806; 31036052; 30446226; 29750542; 29158470; 27637889; 29229854; 28214639; 28912136; 28129467; 26597133; 27077807; 26880763; 27496321; 27532609; 24141111; 23403306; 22522623; 22842821; 21226949; 22106296	30710284; 30868407; 31445888; 31623154; 30885944; 29556233; 30045697; 28935867; 28807938; 29069865; 28921916; 28247948; 28530639; 27793047; 27278086; 27098123; 27127879; 26497012; 25171474; 24798676; 24039846; 21911392; 21169409; 21195708
76	Eosinophilic disorder	29611236; 22981791	30569582
77	Anaplastic thyroid carcinoma	31512776	30196299; 27802204; 16265347
78	Childhood Leukemia	2295067	18452107
79	Tumor Cell Invasion	28317173; 25486572	30893511; 30885944; 30710284; 30970087; 30152542; 30868407; 29400663; 29679610; 29383839; 30196299; 29282275; 28247948; 27922683; 29218245; 28209128; 27862098; 27927689; 28423501; 27802204; 27098123; 27348205; 27127879; 26928771; 26292668; 26744878
80	Hepatitis B	31172579; 31201869 ;30930359; 29524530; 28373196; 28551415; 26315138; 25884105; 21721030; 18083266; 15994231; 8244255; 8340055	28688902; 20026004
81	Steatohepatitis	29855540	31321478

82	Tumor Angiogenesis	22071966; 20379224; 11948141; 10029078	30450809; 23929008
83	Malignant neoplasm of colon and/or rectum	23403306; 19564073	31045759; 31581665; 28775774; 27750112; 25252758; 23783026; 21837681; 21354054; 21218086; 19513503; 16097057
84	Secondary malignant neoplasm of liver	28008721; 21958207; 14977858; 12405292; 11435460	2287817;5 21403839; 17786304; 12239456; 11875708
85	Skin lesion	28752785; 29459858	19703228
86	Malignant neoplasm of endometrium	27532609; 21868529; 21226949	29901152; 19506550
87	Primary malignant neoplasm of lung	30683475; 30622322; 30446226; 26351076; 25486572; 19893592; 19564073; 2560625	30885944; 27127879; 24732359
88	Hepatitis B, Chronic	9024450; 8340055	28688902
89	Neoplasms	31512776; 31776268; 30683475; 31070806; 31627888; 30848981; 31036052; 30393160; 31358054; 31431457; 30675668; 30679806; 30622322; 29750542; 30012853; 29849115; 28912136; 28337378; 28514874; 27188205; 28578991; 28098415; 28483787; 27399807; 26880763	30745574; 31844158; 30868407; 31611961; 29741811; 30111999; 29212855; 29449540; 29400663; 30196299; 30097176; 30221056; 28807938; 28423501; 28832662; 28687621; 28808805; 28244731; 28058579; 27802204; 27750112; 25854377; 24469032; 25880590
90	Solid Neoplasm	9826714; 7776650; 2295067	24037664; 19194111
91	Malignant tumor of cervix	10521926	28921916
92	Degenerative polyarthritis	23369825	17328050; 17105852; 16948116; 16948116
93	Squamous cell carcinoma of esophagus	23639140; 21452064	25217321; 24945657; 22458600; 22408350; 21603862
94	Tumor Progression	31821828; 28849002	31467231; 31474194; 30927479; 29497991; 30111999; 29578167; 29789685; 29204268; 29069865; 27750112; 27278086; 26928771; 25677816; 24469032; 24378831; 22262311; 21837681; 21360024; 20177948; 20019188; 18651563; 18781956; 15900299; 16367903; 16265347
95	Eosinophilia	29611236; 22981791	30569582
96	Nasopharyngeal carcinoma	31312900; 31295651; 29581840	26292668
97	Adenocarcinoma of large intestine	27188205	12239456; 10952782
98	Central neuroblastoma	27637889; 26887385; 28882441; 26943965; 25920530; 25086905; 18726131	28687621; 15852272; 11431772

99	Carcinoma	22555508	27862098; 19194111; 19365673; 11786397; 11875708; 10738249; 9881667; 9072554; 9815629
100	Conventional (Clear Cell) Renal Cell Carcinoma	22344395; 18695887; 15546502; 12631618	22613408; 19365673
101	Pancreatic Neoplasm	20230221	25677816; 19653048; 18350635; 17638890; 16865243
102	Colorectal Carcinoma	23403306; 19564073	31581665; 31045759; 30221056; 29544454; 29383839; 28775774; 28209128; 27053631; 28423501; 27750112; 24936148; 25252758; 23783026; 21837681; 20191297; 21354054; 21218086; 19513503; 16097057
103	Hepatitis	30594597; 22106296	31321478; 28688902
104	Cardiomyopathy, Familial Idiopathic	20350703	19653048

Table S5. List of the human diseases associated with IFN-β and S100A4 protein, according to Open Targets Platform database (https://www.opentargets.org), and corresponding association scores (https://docs.targetvalidation.org/getting-started/scoring). Diseases with association scores for the both proteins exceeding 0.1 are highlighted in yellow.

№	*Disease*	*IFN-β*	*S100A4*
1	B-cell non-Hodgkins lymphoma	0.063	0.014
2	chronic hepatitis B virus infection	0.055	0.014
3	liver neoplasm	0.113	0.1
4	shock	0.044	0.039
5	hepatitis	0.057	0.029
6	ulcerative colitis	0.05	0.014
7	acute myeloid leukemia	0.035	0.062
8	inflammatory bowel disease	0.119	0.074
9	obesity	0.198	0.081
10	pancreatic carcinoma	0.047	0.066
11	Ewing sarcoma	0.045	0.193
12	proteinuria	0.072	0.013
13	medulloblastoma	0.042	0.036
14	cardiac hypertrophy	0.011	0.027
15	lymphangioleiomyomatosis	0.053	0.01
16	weight loss	0.022	0.014
17	lung neoplasm	0.647	0.164
18	immunophenotype	0.03	0.015
19	colitis	0.119	0.068
20	adenocarcinoma	0.783	0.142
21	renal cell adenocarcinoma	0.078	0.084
22	epilepsy	0.288	0.028
23	hyperplasia	0.051	0.026
24	dysplasia	0.029	0.046
25	necrotizing enterocolitis	0.049	0.01
26	metastatic melanoma	0.05	0.012
27	embryonic lethality	0.011	0.013
28	lung cancer	0.647	0.164
29	fibrosarcoma	0.052	0.009
30	triple-negative breast cancer	0.039	0.033
31	cutaneous melanoma	0.038	0.038
32	dilatation	0.01	0.019
33	lymph node metastatic carcinoma	0.029	0.066
34	systemic scleroderma	0.019	0.072

35	pulmonary fibrosis	0.033	0.103
36	acute promyelocytic leukemia	0.012	0.011
37	non-alcoholic fatty liver disease	0.019	0.035
38	cervical cancer	0.047	0.05
39	colorectal adenocarcinoma	0.045	0.064
40	infectious disease	0.295	0.219
41	fever	0.065	0.045
42	asthma	0.095	0.074
43	breast neoplasm	0.716	0.242
44	gastric adenocarcinoma	0.026	0.033
45	nervousness	0.066	0.035
46	increased inflammatory response	0.057	0.073
47	mucous membrane pemphigoid	0.041	0.07
48	viral disease	0.222	0.044
49	central nervous system cancer	0.68	0.124
50	calcinosis	0.027	0.01
51	hepatic fibrosis	0.042	0.01
52	cystic fibrosis	0.027	0.015
53	nervous system injury	0.059	0.061
54	Duchenne muscular dystrophy	0.033	0.048
55	cervical carcinoma	0.033	0.016
56	refractory drug response	0.025	0.041
57	stomach neoplasm	0.064	0.103
58	atherosclerosis	0.052	0.01
59	nicotine dependence	0.06	0.018
60	primitive neuroectodermal tumor	0.083	0.063
61	arthritis	0.13	0.09
62	neurodegeneration	0.048	0.016
63	arterial disorder	0.104	0.078
64	immune system disease	0.36	0.106
65	pulmonary arterial hypertension	0.102	0.076
66	idiopathic pulmonary fibrosis	0.031	0.1
67	osteoarthritis	0.043	0.049
68	eosinophilia	0.031	0.014
69	systemic lupus erythematosus	0.063	0.027
70	breast cancer	0.716	0.242
71	anaplastic astrocytoma	0.045	0.011
72	diabetes mellitus	0.05	0.03
73	leukemia	0.067	0.067

74	non-alcoholic steatohepatitis	0.016	0.01
75	anxiety disorder	0.029	0.011
76	urinary bladder cancer	0.06	0.074
77	polymyositis	0.072	0.024
78	non-small cell lung carcinoma	0.646	0.159
79	spinal cord injury	0.052	0.051
80	liposarcoma	0.01	0.032
81	retinopathy	0.31	0.048
82	liver disease	0.115	0.107
83	breast adenocarcinoma	0.029	0.047
84	head and neck malignant neoplasia	0.078	0.086
85	neuropathy	0.347	0.206
86	prostate carcinoma	0.073	0.129
87	neoplasm	0.85	0.351
88	colon adenocarcinoma	0.016	0.049
89	injury	0.075	0.078
90	panniculitis	0.043	0.012
91	skin neoplasm	0.141	0.261
92	fatty liver disease	0.02	0.046
93	peripheral demyelination	0.06	0.014
94	sepsis	0.155	0.012
95	psoriasis	0.056	0.059
96	lung disease	0.65	0.166
97	hepatoerythropoietic porphyria	0.017	0.087
98	pancreatic ductal adenocarcinoma	0.011	0.053
99	esophageal carcinoma	0.529	0.086
100	amyloidosis	0.013	0.037
101	endometrial carcinoma	0.061	0.089
102	exstrophy-epispadias complex	0.019	0.014
103	adrenal cortex carcinoma	0.029	0.003
104	thyroid gland undifferentiated (anaplastic) carcinoma	0.013	0.048
105	hypoxia	0.046	0.083
106	Kaposi's sarcoma	0.05	0.193
107	keloid	0.011	0.042
108	hemangioma	0.046	0.017
109	head and neck squamous cell carcinoma	0.053	0.071
110	dermatomyositis	0.071	0.021
111	basal cell carcinoma	0.024	0.027
112	rheumatoid arthritis	0.063	0.092

113	osteosarcoma	0.044	0.093
114	Crohn's disease	0.037	0.038
115	renal carcinoma	0.199	0.086
116	glomerulonephritis (disease)	0.048	0.016
117	squamous cell carcinoma	0.055	0.088
118	nervous system disease	0.85	0.273
119	fibrosis	0.063	0.11
120	intraepithelial neoplasia	0.013	0.019
121	Neu-Laxova syndrome	0.02	0.014
122	complication	0.078	0.014
123	clear cell renal carcinoma	0.024	0.041
124	HIV infection	0.058	0.004
125	heart disease	0.283	0.083
126	carcinoma	0.839	0.264
127	bladder transitional cell carcinoma	0.018	0.017
128	melanoma	0.141	0.082
129	chronic obstructive pulmonary disease	0.087	0.071
130	lymphoma	0.067	0.023
131	carcinoma, Lewis lung	0.029	0.035
132	lung carcinoma	0.646	0.164
133	osteopenia	0.012	0.029
134	glioma	0.788	0.13
135	substance dependence	0.062	0.02
136	type II hypersensitivity reaction disease	0.261	0.095
137	kidney neoplasm	0.199	0.088
138	prostate adenocarcinoma	0.072	0.129
139	osteoporosis	0.05	0.073
140	ovarian carcinoma	0.697	0.138
141	vascular disease	0.228	0.212
142	tuberculosis	0.149	0.035
143	severe cutaneous adverse reaction	0.05	0.046
144	toxic encephalopathy	0.025	0.011
145	neuroblastoma	0.082	0.059
146	urethral intrinsic sphincter deficiency	0.043	0.069
147	inflammation	0.102	0.068
148	Alzheimer's disease	0.044	0.004
149	metastatic neoplasm	0.05	0.045
150	esophageal squamous cell carcinoma	0.04	0.085
151	amyotrophic lateral sclerosis	0.036	0.068

152	invasive breast ductal carcinoma	0.009	0.046
153	brain neoplasm	0.788	0.132
154	pancreatitis	0.031	0.029
155	malignant mesothelioma	0.044	0.029
156	chronic myelogenous leukemia	0.04	0.008
157	myeloid leukemia	0.043	0.062
158	colorectal carcinoma	0.063	0.208
159	lung adenocarcinoma	0.642	0.012
160	brain injury	0.045	0.032
161	pleural tuberculosis	0.015	0.031
162	gastric carcinoma	0.042	0.085
163	cancer	0.839	0.346
164	ascites	0.051	0.006
165	experimental autoimmune encephalomyelitis	0.105	0.036
166	hepatocellular carcinoma	0.112	0.097
167	necrosis	0.064	0.019
168	infectious mononucleosis	0.087	0.012
169	heart failure	0.015	0.017
170	relapsing-remitting multiple sclerosis	0.11	0.006
171	hepatitis B virus infection	0.064	0.038
172	colon carcinoma	0.06	0.087
173	non-small cell lung adenocarcinoma	0.02	0.055
174	malignant pancreatic neoplasm	0.058	0.208
175	oral mucositis	0.058	0.012
176	oral squamous cell carcinoma	0.01	0.053
177	vulvar intraepithelial neoplasia	0.038	0.008
178	myocardial infarction	0.024	0.047
179	cholangiocarcinoma	0.01	0.074
180	allergy	0.04	0.043
181	astrocytoma	0.676	0.123
182	nasopharyngeal squamous cell carcinoma	0.047	0.028
183	pneumonia	0.06	0.101
184	periodontitis	0.013	0.057
185	ovarian neoplasm	0.697	0.13
186	myositis	0.076	0.086
187	childhood acute myeloid leukemia	0.013	0.028
188	tongue neoplasm	0.043	0.016
189	cirrhosis of liver	0.061	0.071
190	invasive carcinoma	0.013	0.053

191	congenital heart disease	0.013	0.013
192	undifferentiated carcinoma	0.048	0.05
193	pancreatic adenocarcinoma	0.047	0.045
194	sarcoidosis	0.081	0.02
195	glioblastoma multiforme	0.676	0.123
196	neurodegenerative disease	0.358	0.076
197	breast carcinoma	0.709	0.197
198	Southeast Asian ovalocytosis	0.011	0.01
199	Becker muscular dystrophy	0.01	0.027
200	lupus nephritis	0.032	0.006
201	myocarditis	0.049	0.025
202	X-linked adrenoleukodystrophy	0.046	0.023

Table S6. List of the human diseases associated with IFN-β and S100A6 protein, according to DisGeNET database (http://www.disgenet.org). PubMed identifiers of the references confirming the protein-disease associations are indicated.

№	*Disease*	*IFN-β*	*S100A6*
1	Primary malignant neoplasm	30864696; 30613977; 31167402; 30679806; 31036052; 30446226; 29750542; 29158470; 27637889; 29229854; 28214639; 28912136; 28129467; 26597133; 27077807; 26880763; 27496321; 27532609; 24141111; 23403306; 22522623; 22842821; 21226949; 22106296	29552203; 28123545; 25120023; 19888321; 18714402; 17970874; 15280928; 10952782
2	Coronary Arteriosclerosis	25882064	28174168
3	Conventional (Clear Cell) Renal Cell Carcinoma	22344395; 18695887; 15546502; 12631618	25120023; 25760073
4	Leukemia, Myelocytic, Acute	17047156	2542700
5	Malignant neoplasm of stomach	22159640	24705642; 22681645; 20581057; 17970874
6	Malignant neoplasm of liver	28008721	24281831
7	Acute lymphocytic leukemia	9922477; 2294436	28646023; 22025528
8	Liver carcinoma	28456632; 23887606; 22790964; 19911974; 18618506; 12395321	24281831; 19048101
9	Malignant neoplasm of ovary	11896621	19888321
10	Glioblastoma	29557060; 27637889; 28098415; 29159280; 28912136; 27194146; 25920530; 26441059; 26397698; 24526161; 23384727; 21805051; 20606645; 19197327; 15455376; 14965379; 1501894; 1998958; 1653364	19074870
11	Bladder Neoplasm	11948141; 2297754	26508026

12	Childhood Acute Lymphoblastic Leukemia	9922477	22025528
13	Adenocarcinoma of large intestine	27188205	12239456; 10952782
14	Secondary malignant neoplasm of lymph node	30672717; 23358428	24705642
15	Colon Carcinoma	29496994; 18035482; 1586975	29534068
16	Malignant tumor of colon	29496994;18035482	29534068
17	Liver and Intrahepatic Biliary Tract Carcinoma	28008721	24281831
18	Malignant neoplasm of prostate	29435019; 26339361; 25075566; 23790156; 22429371; 17088978; 16989575; 12231540; 11498771	16015609; 15280928
19	Neuroblastoma	27637889; 26887385; 28882441; 26943965; 25920530; 25086905; 18726131; 16115947	7576953; 1998963
20	Central neuroblastoma	27637889; 26887385; 28882441; 26943965; 25920530; 25086905; 18726131	7576953; 1998963
21	Precursor Cell Lymphoblastic Leukemia Lymphoma	2294436	28646023
22	Hepatitis B	31172579; 31201869; 30930359; 29524530; 28551415; 26315138; 25884105; 21721030; 18083266; 15994231; 8244255; 8340055	20026004
23	Non-Small Cell Lung Carcinoma	30622322; 26431376	18620780
25	Malignant neoplasm of urinary bladder	11948141; 2297754	26508026

24	Secondary Neoplasm	20197756	25120023; 26252518;23007696; 16015609; 9291441; 8261423
25	Alzheimer's Disease	30610591; 31152860 ;31233762; 30076830; 30222725; 24262201	31440382; 15590066
26	Pancreatic Neoplasm	20230221	16278400; 12750293
27	Adult Medulloblastoma	28559152; 24584142	17579622
28	Carcinoma of lung	30622322; 31428903; 30683475; 30446226; 26351076; 25486572; 19893592;19564073; 2560625	31497188; 18620780
29	Meningioma	27873050	15492810
30	Carcinoma of bladder	11948141	26508026
31	Malignant neoplasm of breast	31167402; 26716512; 27077807; 25884794; 24141111; 18608205; 18608205	28051137; 22997041
32	Secondary malignant neoplasm of liver	28008721; 21958207; 14977858; 12405292; 11435460	12239456;10952782
33	Childhood Medulloblastoma	28559152; 24584142	17579622; 17579622
34	Malignant Neoplasms	30864696; 30679806; 31036052; 31167402 ;30613977; 30446226; 29158470; 29750542; 28214639; 26597133; 29229854; 28912136; 28129467; 27637889; 27532609; 26880763; 27496321; 27077807; 24141111; 23403306; 22522623; 22842821; 22106296; 21226949	29544367; 29552203; 28075439; 28123545; 25120023; 24281831; 19888321; 18714402; 17970874; 15280928; 10952782
35	Medulloblastoma	28559152; 24584142	17579622; 17579622
36	Adult Acute Lymphocytic Leukemia	9922477	22025528
37	Amyloidosis	30222725	31440382
38	Myeloid Leukemia, Chronic	30078654; 7544849	2542700
39	Pancreatic carcinoma	23807450	26044826; 25799022

40	Colorectal Carcinoma	23403306; 19564073	24378749
41	Glioblastoma Multiforme	29557060; 29159280; 28912136; 28098415; 27637889 ;27194146; 25920530; 26441059; 24526161; 23384727; 20606645; 19197327; 18714312; 14965379; 1653364; 1998958	19074870
42	Prostate carcinoma	29435019; 26339361; 25075566; 23790156; 22429371; 16989575; 17088978; 12231540; 11498771	16015609; 15280928
43	Tuberculosis	29255077; 29504148; 29017282 28422568; 29258190	27882322; 19912616 11574597
44	Primary malignant neoplasm of lung	30683475; 30622322; 30446226; 26351076; 25486572; 19893592; 19564073; 2560625	31497188; 18620780
45	Melanoma	30864696; 30213181; 31070806; 28649738; 27677689; 28624449; 28877249; 29136453; 27399807; 26102027; 26054674; 25728676; 23370279; 23358428; 23018621; 22555508; 21723255; 21846298; 21493591; 18945721; 16505900; 16928243; 16343929; 12096926	28433799; 9925766; 9716033 9291441; 7654229; 8261423; 1737392
46	Neoplasms, Experimental	2297754	19888321
47	Malignant neoplasm of lung	30683475; 30622322; 30446226; 26351076; 25486572; 19893592; 19564073; 2560625	31497188; 18620780
48	Fibrosis, Liver	25715168	29800549
49	Neoplasm Metastasis	31070806; 30213181; 29202279; 28129467; 26716512; 26102027; 20197756; 12405292; 11948141; 11498771; 10029078; 9815626;	25120023; 26252518; 24705642; 24281831; 23007696; 22681645; 20581057; 18612712; 16015609; 16157226; 12239456; 10952782; 9291441; 7654229; 8261423
50	Childhood Neuroblastoma	26887385; 28882441; 27637889; 26943965; 25920530; 25086905; 18726131	7576953; 1998963

51	Adult Liver Carcinoma	28008721	24281831
52	Tumor Cell Invasion	29618645; 28317173; 25486572	29544367; 29629840;25799022; 25120023; 24705642; 10952782
53	Nasopharyngeal carcinoma	31312900; 31295651; 29581840	27596819
54	Neoplasms	31512776; 31776268 ;30683475; 31070806; 31627888; 30848981; 31036052; 30393160; 31358054; 31431457; 30675668; 30679806; 30622322; 29750542; 30012853; 29849115; 28912136; 28337378; 28514874; 27188205; 28578991; 28098415; 28483787; 27399807; 26880763	31497188; 29629840; 28075439; 28612211; 25760073; 25120023; 26252518; 24281831; 24705642; 24583627; 24217649; 22681645; 19888321; 18620780; 16464796; 12859951; 9291441; 7654229
55	Malignant neoplasm of pancreas	23807450	25799022
56	Tumor Progression	31821828; 28849002	22681645; 8261423
57	Stomach Carcinoma	22159640	26044826; 24705642; 22681645; 20581057; 17970874
58	Breast Carcinoma	31167402; 27207649; 27077807; 26716512; 25884794; 24141111; 18608205 ;18608205; 9059994	28051137; 22997041; 9716033

Table S7. List of the human diseases associated with IFN-β and S100A6 protein, according to Open Targets Platform database (https://www.opentargets.org), and corresponding association scores (https://docs.targetvalidation.org/getting-started/scoring). Diseases with association scores for the both proteins exceeding 0.1 are highlighted in yellow.

№	*Disease*	*IFN-β*	*S100A6*
1	breast adenocarcinoma	0.029	0.049
2	legionnaires' disease	0.039	0.01
3	complication	0.078	0.01
4	Duchenne muscular dystrophy	0.033	0.035
5	allergy	0.04	0.011
6	multiple myeloma	0.037	0.034
7	nervous system disease	0.85	0.15
8	hyperglycemia	0.012	0.011
9	epilepsy	0.288	0.044
10	heart failure	0.015	0.034
11	pulmonary arterial hypertension	0.102	0.013
12	cognitive disorder	0.284	0.031
13	stomach neoplasm	0.064	0.091
14	cervical cancer	0.047	0.05
15	severe cutaneous adverse reaction	0.05	0.013
16	pancreatic ductal adenocarcinoma	0.011	0.058
17	cirrhosis of liver	0.061	0.043
18	Alzheimer's disease	0.044	0.03
19	lung neoplasm	0.647	0.102
20	lymph node metastatic carcinoma	0.029	0.035
21	squamous cell carcinoma	0.055	0.048
22	non-small cell lung carcinoma	0.646	0.097
23	prostate intraepithelial neoplasia	0.01	0.027
24	potassium-aggravated myotonia	0.044	0.02
25	pleural effusion	0.042	0.022
26	diabetes mellitus	0.05	0.016
27	nervousness	0.066	0.038
28	lung carcinoma	0.646	0.101
29	melanoma	0.141	0.073
30	idiopathic pulmonary fibrosis	0.031	0.01
31	carcinoma	0.839	0.115
32	cancer	0.839	0.116
33	ovarian neoplasm	0.697	0.067
34	osteosarcoma	0.044	0.08

35	glioblastoma multiforme	0.676	0.038
36	Friedreich ataxia	0.011	0.005
37	hypothyroidism	0.139	0.011
38	edema	0.023	0.014
39	systemic scleroderma	0.019	0.03
40	dilated cardiomyopathy	0.257	0.029
41	colon carcinoma	0.06	0.035
42	meningioma	0.041	0.037
43	clear cell renal carcinoma	0.024	0.036
44	colorectal adenocarcinoma	0.045	0.061
45	pain	0.044	0.01
46	ischemia reperfusion injury	0.023	0.027
47	nicotine dependence	0.06	0.004
48	ischemia	0.053	0.018
49	sepsis	0.155	0.037
50	adrenal cortex carcinoma	0.029	0.007
51	small cell lung carcinoma	0.033	0.017
52	leukemia	0.067	0.065
53	intraepithelial neoplasia	0.013	0.032
54	invasive breast ductal carcinoma	0.009	0.027
55	chronic bronchitis	0.01	0.019
56	lung disease	0.65	0.103
57	bladder tumor	0.061	0.035
58	astrocytoma	0.676	0.025
59	exhaustion	0.016	0.011
60	nasopharyngeal squamous cell carcinoma	0.047	0.035
61	urinary system disease	0.774	0.084
62	immunophenotype	0.03	0.02
63	cutaneous melanoma	0.038	0.036
64	uveal melanoma	0.037	0.02
65	neurodegenerative disease	0.358	0.044
66	infectious disease	0.295	0.033
67	prostate adenocarcinoma	0.072	0.066
68	proteinuria	0.072	0.015
69	endometrial carcinoma	0.061	0.038
70	basal cell carcinoma	0.024	0.024
71	fibrosis	0.063	0.054
72	cholangiocarcinoma	0.01	0.074
73	hepatoblastoma	0.015	0.033

74	pulmonary fibrosis	0.033	0.027
75	mental deterioration	0.019	0.012
76	non-small cell lung adenocarcinoma	0.02	0.02
77	bronchoalveolar adenocarcinoma	0.012	0.026
78	colorectal carcinoma	0.063	0.094
79	tuberculosis	0.149	0.01
80	infertility	0.203	0.021
81	chronic obstructive pulmonary disease	0.087	0.04
82	hepatoerythropoietic porphyria	0.017	0.014
83	bronchioloalveolar carcinoma	0.058	0.014
84	leiomyosarcoma	0.014	0.016
85	obesity	0.198	0.01
86	injury	0.075	0.06
87	shock	0.044	0.061
88	pancreatic carcinoma	0.047	0.037
89	Southeast Asian ovalocytosis	0.011	0.01
90	dysplasia	0.029	0.016
91	fatigue	0.066	0.013
92	lung cancer	0.647	0.102
93	adenocarcinoma	0.783	0.098
94	hyperplasia	0.051	0.027
95	renal cell adenocarcinoma	0.078	0.037
96	vasculitis	0.043	0.01
97	sarcoma	0.074	0.082
98	neoplasm	0.85	0.117
99	hepatocellular carcinoma	0.112	0.087
100	amyloidosis	0.013	0.059
101	ascites	0.051	0.055
102	systemic lupus erythematosus	0.063	0.013
103	sarcoidosis	0.081	0.01
104	encephalomyelitis	0.226	0.026
105	necrosis	0.064	0.015
106	urinary bladder cancer	0.06	0.033
107	fetal growth restriction	0.012	0.011
108	malignant pancreatic neoplasm	0.058	0.094
109	Becker muscular dystrophy	0.01	0.015
110	liver neoplasm	0.113	0.095
111	myocardial infarction	0.024	0.045
112	breast carcinoma	0.709	0.051

113	systemic autoimmune disease	0.08	0.035
114	infectious mononucleosis	0.087	0.011
115	breast neoplasm	0.716	0.056
116	melanocytic nevus	0.027	0.03
117	acute kidney failure	0.01	0.031
118	Exstrophy-epispadias complex	0.019	0.017
119	kidney neoplasm	0.199	0.037
120	oral squamous cell carcinoma	0.01	0.026
121	glioma	0.788	0.025
122	medulloblastoma	0.042	0.037
123	pancreatitis	0.031	0.024
124	acute lymphoblastic leukemia	0.065	0.061
125	ovarian carcinoma	0.697	0.076
126	amyotrophic lateral sclerosis	0.036	0.041
127	Neu-Laxova syndrome	0.02	0.034
128	stroke	0.084	0.01
129	neuroblastoma	0.082	0.052
130	cervical carcinoma	0.033	0.011
131	kidney disease	0.297	0.046
132	cardiac hypertrophy	0.011	0.041
133	Graves disease	0.012	0.01
134	hypersensitivity reaction disease	0.261	0.036
135	relapsing-remitting multiple sclerosis	0.11	0.006
136	endometrium adenocarcinoma	0.024	0.024
137	hypertension	0.102	0.064
138	respiratory failure	0.067	0.01
139	paraneoplastic neurologic syndrome	0.01	0.012
140	hypoxia	0.046	0.023
141	anaplastic astrocytoma	0.045	0.011
142	brain injury	0.045	0.032
143	breast cancer	0.716	0.056
144	undifferentiated carcinoma	0.048	0.018
145	inflammation	0.102	0.016
146	urethral intrinsic sphincter deficiency	0.043	0.012
147	metastatic neoplasm	0.05	0.021
148	substance dependence	0.062	0.014
149	B-cell non-Hodgkins lymphoma	0.063	0.018
150	gastric carcinoma	0.042	0.017

Table S8. List of the human diseases associated with IFN-β, S100A1, S100A4 and S100A6 proteins, according to DisGeNET database (http://www.disgenet.org).

№	*Disease*
1	Malignant neoplasm of breast
2	Breast Carcinoma
3	Malignant neoplasm of urinary bladder
4	Bladder Neoplasm
5	Malignant Neoplasms
6	Neoplasm Metastasis
7	Neoplasms
8	Primary malignant neoplasm
9	Malignant neoplasm of lung
10	Liver carcinoma
11	Conventional (Clear Cell) Renal Cell Carcinoma
12	Non-Small Cell Lung Carcinoma
13	Tumor Cell Invasion
14	Tumor Progression
15	Secondary malignant neoplasm of lymph node
16	Malignant neoplasm of stomach
17	Malignant neoplasm of pancreas
18	Malignant neoplasm of ovary

Table S9. List of the human diseases associated with IFN-β, S100A1, S100A4 and S100A6 proteins, according to Open Targets Platform database (https://www.opentargets.org), and corresponding association scores (https://docs.targetvalidation.org/getting-started/scoring). Diseases with association scores for the proteins exceeding 0.1 are highlighted in yellow.

№	*Disease*	*IFN-β*	*S100A1*	*S100A4*	*S100A6*
1	adenocarcinoma	0.783	0.383	0.142	0.098
2	Alzheimer's disease	0.044	0.04	0.004	0.03
3	amyloidosis	0.013	0.024	0.037	0.059
4	anaplastic astrocytoma	0.045	0.011	0.011	0.011
5	astrocytoma	0.676	0.015	0.123	0.025
6	breast adenocarcinoma	0.029	0.011	0.047	0.049
7	breast cancer	0.716	0.046	0.242	0.056
8	cancer	0.839	0.538	0.346	0.116
9	carcinoma	0.839	0.537	0.264	0.115
10	cardiac hypertrophy	0.011	0.041	0.027	0.041
11	cervical cancer	0.047	0.036	0.05	0.05
12	chronic obstructive pulmonary disease	0.087	0.012	0.071	0.04
13	cirrhosis of liver	0.061	0.2	0.071	0.043
14	clear cell renal carcinoma	0.024	0.013	0.041	0.036
15	colorectal carcinoma	0.063	0.011	0.208	0.094
16	complication	0.078	0.011	0.014	0.01
17	cutaneous melanoma	0.038	0.02	0.038	0.036
18	dysplasia	0.029	0.017	0.046	0.016
19	endometrial carcinoma	0.061	0.031	0.089	0.038
20	glioblastoma multiforme	0.676	0.031	0.123	0.038
21	glioma	0.788	0.021	0.13	0.025
22	heart failure	0.015	0.303	0.017	0.034
23	hyperplasia	0.051	0.018	0.026	0.027
24	hypoxia	0.046	0.064	0.083	0.023
25	idiopathic pulmonary fibrosis	0.031	0.193	0.1	0.01
26	infectious disease	0.295	0.198	0.219	0.033
27	inflammation	0.102	0.061	0.068	0.016
28	injury	0.075	0.036	0.078	0.06
29	kidney neoplasm	0.199	0.066	0.088	0.037
30	lung carcinoma	0.646	0.424	0.164	0.101
31	lymph node metastatic carcinoma	0.029	0.037	0.066	0.035
32	malignant pancreatic neoplasm	0.058	0.011	0.208	0.094
33	medulloblastoma	0.042	0.062	0.036	0.037
34	melanoma	0.141	0.047	0.082	0.073

35	myocardial infarction	0.024	0.07	0.047	0.045
36	necrosis	0.064	0.012	0.019	0.015
37	neoplasm	0.85	0.586	0.351	0.117
38	nervous system disease	0.85	0.322	0.273	0.15
39	nervousness	0.066	0.018	0.035	0.038
40	neuroblastoma	0.082	0.01	0.059	0.052
41	nicotine dependence	0.06	0.013	0.018	0.004
42	non-small cell lung carcinoma	0.646	0.004	0.159	0.097
43	obesity	0.198	0.02	0.081	0.01
44	ovarian carcinoma	0.697	0.082	0.138	0.076
45	pulmonary arterial hypertension	0.102	0.309	0.076	0.013
46	renal cell adenocarcinoma	0.078	0.061	0.084	0.037
47	sepsis	0.155	0.007	0.012	0.037
48	shock	0.044	0.016	0.039	0.061
49	squamous cell carcinoma	0.055	0.43	0.088	0.048
50	stomach neoplasm	0.064	0.368	0.103	0.091
51	substance dependence	0.062	0.027	0.02	0.014
52	urethral intrinsic sphincter deficiency	0.043	0.018	0.069	0.012
53	urinary bladder cancer	0.06	0.049	0.074	0.033

Table S10. List of the human diseases associated with IFN-β, S100A1, S100A4, S100A6 and S100P proteins, according to Open Targets Platform database (https://www.opentargets.org), and corresponding association scores (https://docs.targetvalidation.org/getting-started/scoring). Diseases with association scores for the proteins exceeding 0.1 are highlighted in yellow.

№	*Disease*	*IFN-β*	*S100A1*	*S100A4*	*S100A6*	*S100P*
1	adenocarcinoma	0.783	0.383	0.142	0.098	0.122
2	cancer	0.839	0.538	0.346	0.116	0.262
3	carcinoma	0.839	0.537	0.264	0.115	0.229
4	colorectal carcinoma	0.063	0.011	0.208	0.094	0.219
5	dysplasia	0.029	0.017	0.046	0.016	0.028
6	hyperplasia	0.051	0.018	0.026	0.027	0.016
7	hypoxia	0.046	0.064	0.083	0.023	0.018
8	inflammation	0.102	0.061	0.068	0.016	0.024
9	lung carcinoma	0.646	0.424	0.164	0.101	0.075
10	lymph node metastatic carcinoma	0.029	0.037	0.066	0.035	0.02
11	medulloblastoma	0.042	0.062	0.036	0.037	0.023
12	melanoma	0.141	0.047	0.082	0.073	0.051
13	myocardial infarction	0.024	0.07	0.047	0.045	0.031
14	neoplasm	0.85	0.586	0.351	0.117	0.299
15	ovarian carcinoma	0.697	0.082	0.138	0.076	0.071
16	pulmonary arterial hypertension	0.102	0.309	0.076	0.013	0.01
17	sepsis	0.155	0.007	0.012	0.037	0.061
18	squamous cell carcinoma	0.055	0.43	0.088	0.048	0.051
